# EPR-Spin Trapping and Flow Cytometric Studies of Free Radicals Generated Using Cold Atmospheric Argon Plasma and X-Ray Irradiation in Aqueous Solutions and Intracellular Milieu

**DOI:** 10.1371/journal.pone.0136956

**Published:** 2015-08-28

**Authors:** Hidefumi Uchiyama, Qing-Li Zhao, Mariame Ali Hassan, Gabor Andocs, Nobuyuki Nojima, Keigo Takeda, Kenji Ishikawa, Masaru Hori, Takashi Kondo

**Affiliations:** 1 Tateyama Machine Co., Ltd., Toyama 930–1305, Japan; 2 Department of Radiological Sciences, Graduate School of Medicine and Pharmaceutical Sciences, University of Toyama, Toyama 930–0194, Japan; 3 Plasma Nanotechnology Research Center Nagoya University, Nagoya 464–8601, Japan; University of Kansas, UNITED STATES

## Abstract

Electron paramagnetic resonance (EPR)-spin trapping and flow cytometry were used to identify free radicals generated using argon-cold atmospheric plasma (Ar-CAP) in aqueous solutions and intracellularly in comparison with those generated by X-irradiation. Ar-CAP was generated using a high-voltage power supply unit with low-frequency excitation. The characteristics of Ar-CAP were estimated by vacuum UV absorption and emission spectra measurements. Hydroxyl (·OH) radicals and hydrogen (H) atoms in aqueous solutions were identified with the spin traps 5,5-dimethyl-1-pyrroline *N*-oxide (DMPO), 3,3,5,5-tetramethyl-1-pyrroline-*N*-oxide (M_4_PO), and phenyl *N*-t-butylnitrone (PBN). The occurrence of Ar-CAP-induced pyrolysis was evaluated using the spin trap 3,5-dibromo-4-nitrosobenzene sulfonate (DBNBS) in aqueous solutions of DNA constituents, sodium acetate, and L-alanine. Human lymphoma U937 cells were used to study intracellular oxidative stress using five fluorescent probes with different affinities to a number of reactive species. The analysis and quantification of EPR spectra revealed the formation of enormous amounts of ·OH radicals using Ar-CAP compared with that by X-irradiation. Very small amounts of H atoms were detected whereas nitric oxide was not found. The formation of ·OH radicals depended on the type of rare gas used and the yield correlated inversely with ionization energy in the order of krypton > argon = neon > helium. No pyrolysis radicals were detected in aqueous solutions exposed to Ar-CAP. Intracellularly, ·OH, H_2_O_2_, which is the recombination product of ·OH, and OCl^-^ were the most likely formed reactive oxygen species after exposure to Ar-CAP. Intracellularly, there was no practical evidence for the formation of NO whereas very small amounts of superoxides were formed. Despite the superiority of Ar-CAP in forming ·OH radicals, the exposure to X-rays proved more lethal. The mechanism of free radical formation in aqueous solutions and an intracellular milieu is discussed.

## Introduction

Plasma medicine is an emerging interdisciplinary field including the technology of low-temperature plasma [1,2]. Despite the novelty of this technology, its potential has been demonstrated in various applications such as sterilization [3,4], blood coagulation [5], wound healing [6,7], cancer treatment [8–12], dentistry [13,14], and regenerative medicine of bone [15,16]. The technology offers many advantages including high efficiency, convenience of operation in terms of design and cost, being a dry procedure, applicability to temperature-sensitive materials, and absence of no hazardous residues. A physical cold plasma is a partially ionized gas generated by flowing a gas through a relatively high electric field. A series of excitations and dissociative collisions with the surrounding gas at or near atmospheric pressure takes place producing a mixture of electrons, negative and positive ions, excited gas species, free radicals, and electromagnetic radiation without heating the surrounding gas. Hence, this type of plasma is called cold atmospheric plasma (CAP) [1–16]. Plasma produces a cocktail of oxygen and nitrogen radicals that can promptly react with all biomolecules that come across their way including living cells. These free radicals are the focus of emerging interests in biomedical CAP applications. In general, free radicals are a double-edged sword that can modify a plethora of biomolecules and cellular pathways wiring a range of functions from proliferation to cell death depending on their composition and dose [17]. The chemical output of CAP can be tuned in order to achieve the best results. As tunability requires knowledge, relentless research has started to pay off in terms of the understanding of some of the mechanisms underlying plasma effects and in defining some reactive species as key players. For example, in bacteriostatic and bactericidal applications, it has been demonstrated that plasma-generated particles and UV photons can modify DNA constituents and induce DNA strand breaks, and inactivate proteins in addition to damage of the cellular envelope [18–21]. In one study, dose-dependent effects were reported to range from enhancement of cell proliferation to induction of apoptosis primarily due to the formation of intracellular reactive oxygen species (ROS). The observed DNA damage is initiated by the production of active neutral species induced *via* organic peroxides in a growth medium [22]. In HeLa cells, nitrogen and air plasma jet induce apoptosis *via* ROS generation and dysfunction of mitochondria, as confirmed by the use of various ROS scavengers, which mitigate apoptosis induction [23]. Hydrogen peroxide (H_2_O_2_) has been shown to play a central role in physical plasma-induced oxidative stress in human blood cells [24]. Endoplasmic stress in response to oxidative and nitrative stresses induced by a CAP jet of helium containing 1% oxygen has been claimed to mediate apoptosis in HepG2 cells [25]. ROS and/or reactive nitrogen species (RNS) generated by plasmas trigger signaling pathways involving JNK and p38, and promote mitochondrial perturbation leading to apoptosis [26]. To date, there has been no unifying concept for the chemical make-up of the output jet and how the different species can modify cellular activities. Owing to the absence of a universal plasma set up and the high variability of operating parameters, extensive efforts are still required in order to gain knowledge and hence control of CAP performance. In this study, we examined the fluxes of reactive moieties generated by a CAP generator developed *in house* with argon gas flow in aqueous solutions and cultured cells using electron paramagnetic resonance (EPR) spin trapping.

EPR-spin trapping is an analytical technique employed in the detection and identification of short-lived free radicals. Spin trapping involves the addition of radicals to nitrone spin traps to form a spin adduct, a nitroxide-based radical of relatively longer half-life to allow its detection using an EPR spectrometer. Depending on the radical, each spin adduct usually yields a distinctive EPR spectrum characteristic to that radical. The spin adduct is usually identified based on the basis of the measurement of hyperfine-coupling constants of relevant nuclei. The relative amount of spin adducts can also be obtained by comparing their spectra with that of a stable nitroxide. In this study, EPR-spin trapping using a number nitrone spin traps was utilized to identify and quantify free radicals in aqueous solutions exposed to Ar-CAP. The obtained EPR spectra were compared with the spectra from radiolysis of air-saturated water. Other radicals that might arise following exposure to Ar-CAP were detected using the nitrone spin trap 3,5-dibromo-4-nitrosobenzene sulfonate (DBNBS) [27,28]. DBNBS is a useful agent for detecting pyrolysis radicals which are formed in high temperature interfacial regions produced by ultrasonic cavitation, e.g., methyl radicals in sodium acetate aqueous solutions [29]. Furthermore, the intracellular oxidative stress induced by Ar-CAP was evaluated by flow cytometry using a number of fluorescent probes to identify intracellular reactive species.

## Materials and Methods

### Chemicals

The inert gases helium (He), neon (Ne), argon (Ar), krypton (Kr), and xenon (Xe) were bought from Hokusan Co., Ltd. (Toyama, Japan). All gases were of pure grade (≥ 99.9%). The spin traps 5,5-dimethyl-1-pyrroline N-oxide (DMPO; Labotec Co., Ltd., Tokyo, Japan), 3,3,5,5-tetramethyl-1-pyrroline-N-oxide (M_4_PO; Sigma Aldrich Chem. Co., MO), phenyl N-t-butylnitrone (PBN; Sigma Aldrich Chem.), 3,5-dibromo-4-nitrosobenzene sulfonate (DBNBS; Wako Pure Chemical Industries, Ltd., Osaka, Japan), and 2-(4-carboxyphenyl)-4,4,5,5-tetramethylimidazoline-1-oxyl-3-oxide (cPTIO; Dojindo, Kumamoto, Japan) were used to detect the presence of different free radical species in ultrapure water following exposure to CAP by EPR spin trapping.

Thymine, thymidine, uracil, uridine, sodium acetate, and L-alanine were obtained from Wako Pure Chemical Industries, Ltd. Hydroxyphenyl fluorescein (HPF), aminophenyl fluorescein (APF), and diaminofluorescein-2 (DAF-2DA) were from Sekisui Medical Co., Ltd. (Tokyo, Japan). Hydroethidine (HE) and 2',7'-dichlorofluorescein diacetate (DCFH-DA) were from Molecular Probes Inc. (Eugene, OR).

### Cells

Human myelomonocytic lymphoma U937 cells were obtained from Human Sciences Research Resource Bank (Japan Human Sciences Foundation, Tokyo, Japan). They were maintained in RPMI-1640 medium (Wako) supplemented with 10% heat-inactivated fetal bovine serum (FBS) at 37°C in humidified air with 5% CO_2_.

### Cold atmospheric argon plasma irradiation system

**Fig 1:**
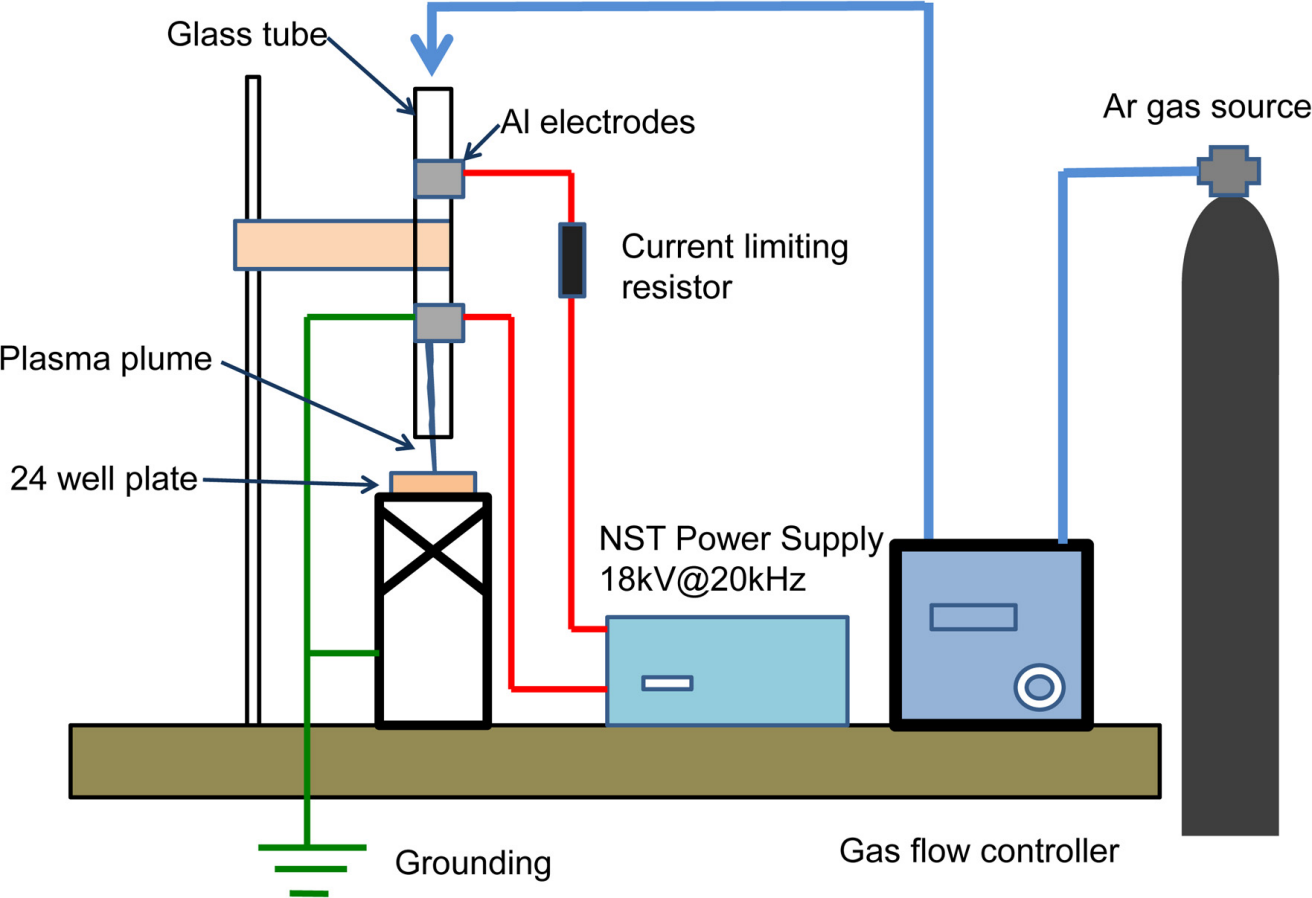
Block diagram of cold atmospheric plasma (CAP) exposure system. Al; aluminum, Ar; argon.

A jet of Ar-plasma was produced in a quartz tube of 2.0 mm inner diameter and about 150 mm length. Two electrodes connected to a high-voltage power supply unit with low frequency (18 kV peak-to-peak, 20 kHz) were fixed on the quartz tube surface. Discharge voltage was measured with a high-voltage probe (P6015A; Tektronik, Inc., Tokyo, Japan). The Ar-gas flow rate was controlled with a gas flow controller (MODEL8500 series; KOFLOC Co., Ltd., Kyoto Japan). The Ar gas flow was at 2 L/min. The length of the plasma jet was approximately 10 mm. The operational parameters for typical experiments were the irradiation distance between the tip of the quartz tube and the solution surface, and irradiation time. The system and the experimental setup are illustrated in Fig 1.

### Temperature measurement of the medium during Ar-CAP irradiation

The temperature changes of the medium during the Ar-CAP irradiation was carried out by a four channel fluoroptic temperature measurement system. (Luxtron m3300 Biomedical Lab Kit, Lumasense Technologies, Santa Clara, CA). The temperature measurement probe is a 0.5 mm in diameter optical fiber, totally insensitive for electromagnetic field. The sample to be measured was 1ml tap water in a well of a 24 well plate. All the four probes of the temperature measurement system were immersed into the water to be irradiated with Ar-Cap, on a bottom of the well, exactly in the way of the plasma plume.

### Optical emissions from Ar-CAP

Optical emissions Ar-CAP were collected by an optic fiber and a lens directed to a position at 8 mm in the remote region of Ar-CAP. The emission were observed using a spectrometer (Shamrock SR-561-B1; grating, 600 l/mm^-1^; ANDOR, Belfast, UK) and an intensified charge-coupled-device (ICCD) camera (iStar DH734-25F-03; ANDOR).

### Atomic radicals generated by Ar-CAP in gas phase

The absolute densities of O (^3^P_j_) and N (^4^S^o^) atoms were measured by vacuum ultraviolet absorption spectroscopy (VUVAS) with a micro discharge hollow cathode lamp (MHCL) as a light source operated at a fixed pressure of 1 atm [30]. The transition lines used for measuring O atoms were 3s ^3^S^o^-2p^4 3^P_2_ at 130.217 nm, 3s ^3^S^o^-2p^4 3^P_1_ at 130.487 nm, and 3s ^3^S^o^-2p^4 3^P_0_ at 130.604 nm. Those used for measuring N atoms were ^4^P_5/2_-^4^S^o^_3/2_ at 119.96 nm, ^4^P_3/2_ -^4^S^o^_3/2_ at 120.02 nm, and ^4^P_1/2_-^4^S^o^_3/2_ at 120.07 nm. The VUV light emitted from the MHCL was transmitted through a measured region with an absorption length of 7 mm set using two MgF_2_ windows attached to the light-guide pipes made of ceramics. The transmitted VUV light was dispersed by a VUV monochromator and detected by a photomultiplier tube. The total absorption intensity for three sublevels of O and N atoms was measured. Ar-CAP was located 8 mm above the measured region. To remove background absorption due to atmospheric gases, N_2_ gas was used as a purge gas to maintain the pressure of the chamber at atmospheric pressure. The absorption intensity of the O_2_ gas feed was measured using the transition lines of H_2_ molecules emitted from MHCL at approximately 128 nm [31]. All optical parts were evacuated at high vacuum using a turbo molecular pump. The slit width of the VUV monochromator was set to 100 μm, and the wavelength resolution was within 0.4 nm.

### Chemical dosimeter

The chemical effects of Ar-CAP were measured by a ferrous-ferric ion (Fricke) dosimeter. Changes in absorbance of the chemical system with exposure time were determined with a spectrophotometer at 304 nm.

### X-ray irradiation

X-ray irradiation was carried out at room temperature using an X-ray generator (MBR-1520R-3, Hitachi Medico Technology, Kashiwa, Japan) operated at 150 kV and 20 mA with a 1 mm Al filter at a dose rate of 5 Gy/min determined by Fricke’s chemical dosimetry. The air-saturated sample solutions (1 ml) in plastic tubes were irradiated on a rotating table at 6 rpm. Immediately after X-irradiation, samples were measured with EPR and UV-spectrophotometer. Final doses were 5, 7.5 and 10 Gy.

### Free radical detection by EPR spin trapping

The detection of ·OH radicals and H atoms induced by Ar-CAP was carried out using the spin traps DMPO, PBN, and M_4_PO. An aqueous solution containing a spin trap at a concentration of 10 mM was treated with CAP at increasing durations and distances. Immediately after irradiation, the samples were transferred to a glass capillary tube (VC-HO75P, Terumo, Tokyo, Japan) and inserted into a special quartz tube in an EPR spectrometer (RFR-30, Radical Research Inc., Tokyo, Japan). In general, EPR setting were microwave power; 4 mW, frequency; 9.425 GHz, center magnetic field; 329.5 mT, and modulation width; 0.1 mT. The nitroso spin trap DBNBS was used at a concentration of 5 mM for the detection of radicals derived from thymine (20 mM), thymidine (100 mM), uracil (20 mM), uridine (20 mM), L-alanine (1 M), and sodium acetate (3 M) aqueous solutions. The EPR spectra of the treated samples were recorded at room temperature. The yields of spin adducts were determined using the stable nitroxide radical 3-carbamoyl-2,2,5,5-tetramethyl-1-pyrroline-1-oxide as a standard. The peak heights of EPR signals were expressed in relative units compared with that of the Mn^2+^ internal standard, one unit was calculated to approximately equal 7.7 x 10^6^ M nitroxide radicals.

### Intracellular oxidative stress measured by flow cytometry

Intracellular oxidative stress following CAP treatment was assessed flow cytometrically using a number of fluorescent probes with different affinities to detect a wide range of free radical species. One million cells were preloaded with a probe in phosphate-buffered saline (PBS) for 15 min at 37°C and then exposed to Ar-CAP. Immediately after the exposure, the cells were injected into the flow cytometer to analyze at least 10,000 events. The probes included were DCFH-DA (10 μM) for overall oxidative stress, HE (10 μM) for superoxide anion (O_2_·^-^), HPF (2.5 μM) for ·OH, and peroxy nitrite (ONOO^-^); APF (2.5 μM) for ·OH, ONOO^-^and hypochlorite (OCl^-^), DAF-2 DA (10 μM) for nitric oxide (NO).

### Apoptosis measurement by Annexin V-FITC/ PI staining

Apoptosis induction following exposure to Ar-CAP and X-rays for different periods and doses, respectively, was investigated flow cytometrically. The irradiated cells were incubated at 37°C for 6 h before they were collected, washed with cold PBS, and suspended in the binding buffer of the Apoptosis Detection Kit (Immunotech, Marseille, France). The cells were then stained with fluorescein isothiocyanate (FITC)-labeled annexin V and propidium iodide (PI) at 4°C for 20 min in the dark according to the manufacturer’s instructions. Finally, samples were injected into the flow cytometer where at least 10,000 events were counted. The dot plots display the percentages of cells externalizing phosphatidylserine bound to FITC-annexin V (early apoptosis), cells with damaged membranes internalizing PI (necrosis), or both (secondary necrosis). The total apoptosis fraction is expressed as the sum of early apoptotic and secondary necrotic fractions.

### Measurement of nitrate/nitrite concentration in aqueous solutions

Briefly, 1 ml milli-Q water was added to 24-well tissue culture plate, and Ar-CAP irradiation was performed for 1 min. After treatment, 80 μL sample was taken out to 96-well plate, and a NO_2_/NO_3_ assay was performed using a NO_2_/NO_3_ Assay Kit-CII (Dojindo, Kumamoto, Japan), the optical density was measured at 570 nm by an automatic microplate reader (Bio-Rad, Hercules, CA).

### Statistical analysis

Experiments were performed in independent triplicates. All results are shown as mean ± standard deviation (SD). Tests of significance were performed using unpaired t-student test (two-tailed) with *p* < 0.05 considered to indicate a statistically significant difference. Pearson’s correlation coefficient was used to test the correlation among obtained data. All tests were performed using GraphPad Prism 5 (GraphPad Software; San Diego, CA).

## Results and Discussion

### 1. Characterization of Ar-CAP

**Fig 2:**
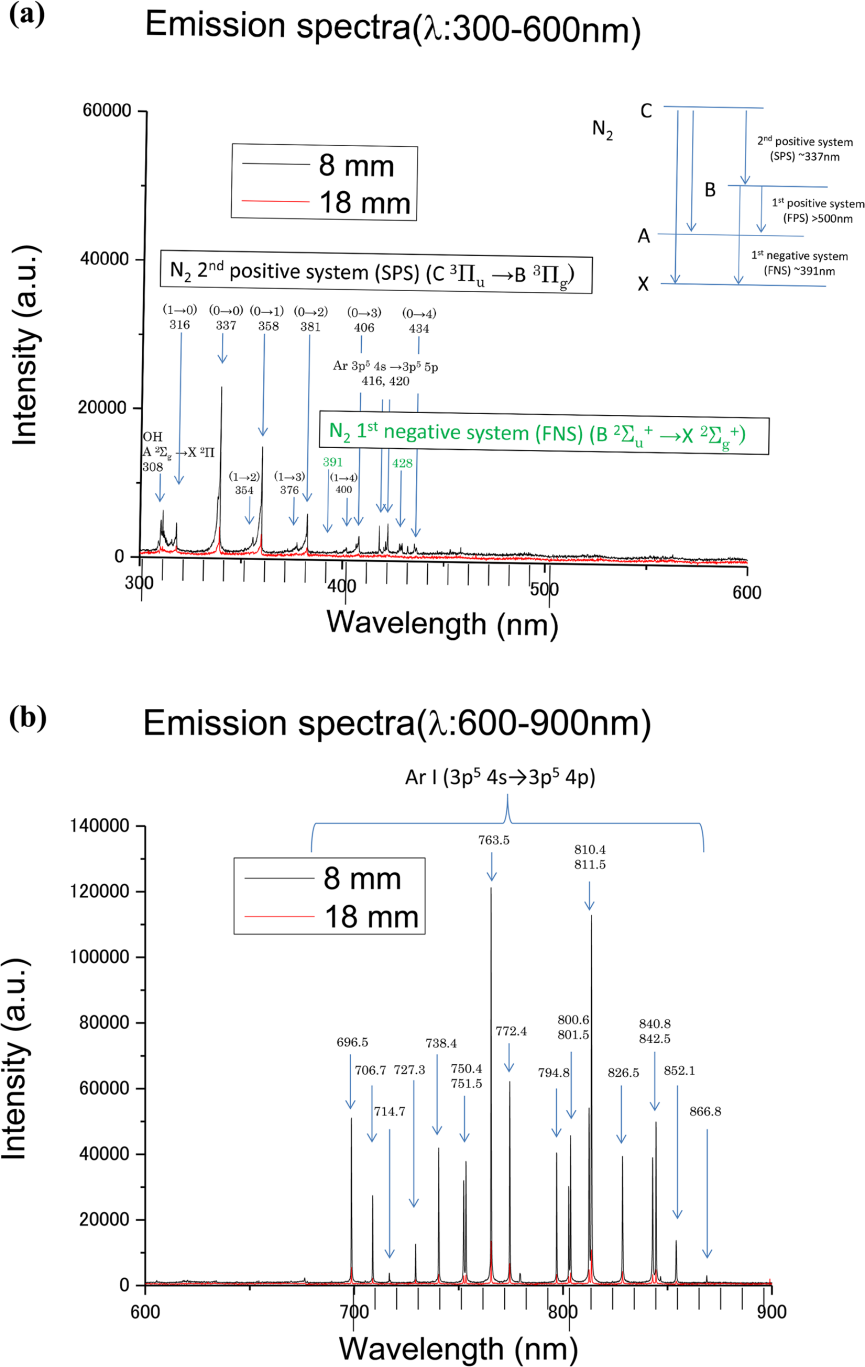
Optical emission and atomic radicals generated by Ar-CAP. Emission spectra (λ: 300–900 nm) of Ar-CAP at distances of 8 and 18 mm. (a) (λ: 300–450 nm), emission spectra from excited N_2_ and ·OH. (b) (λ: 700–850 nm), emission spectra from excited states of Ar.

Optical emission spectra were measured to obtain the spectral lines emitted from excited-state species in Ar-CAP. Fig 2A and 2B show typical optical emission spectra from Ar-CAP ranging from 300 to 900 nm at distances of 8 and 18 mm. Because only Ar gas was fed into the plasma generator, most of the emission lines and bands observed between 700 and 850 nm were mainly attributed to the excited states of Ar. Typically, the emission lines from excited Ar (3p^5^ 4s→3p^5^ 4p) were observed at 696.5, 706.7, 727.3, 738.4, 750.4, 751.5, 763.5, 772.4, 794.8, 800.6, 801.5, 810.4, 811.5, 826.5, 840.8, 842.5, and 852.1 nm. In addition, between 300 and 450 nm, the emission bands from excited N_2_ were observed at approximately 316, 337, 358, 381, 406, and 416 nm for the N_2_ 2^nd^ positive system (C ^3^Π_u_→B ^3^Π_g_). The emission band signals for the N_2_^+^ 1^st^ negative system (B ^2^Σ_u_+→X ^2^Σ_g_^+^) appeared relatively weak in the range between 391 and 428 nm. An emission band from ·OH (A ^2^Σ_g_→ X ^2^Π) occurred with a relatively low signal intensity at approximately 308 nm. Because the emission bands of N_2_ and ·OH were also detected, these findings indicate that the effluent Ar gas mixed with atmospheric air at remote regions beyond the exit of the glass tube. Optical emission spectroscopy is essentially more sensitive for measuring electron temperature than for measuring the densities of the observed species. Thus, the optical emission data do not enable the quantitative estimation of the density of any species.

Furthermore, the absolute densities of atomic species were measured by VUVAS. The average numbers of O atoms were 6.3×10^13^ cm^-3^ at 8 mm distance, 4.5×10^13^ cm^-3^ at 13 mm, and 2.3×10^13^ cm^-3^ at 18 mm. The density of O atoms thus correlates indirectly with the distance from the plasma tube nozzle and the O atoms fed into the samples at remote regions can be calculated using the first-order kinetics within the distance range tested. Assuming that the gas is at equilibrium at room temperature, then the O atom fluxes would be in the order of 10^17^ cm^-2^ s^-1^, and the total O atom dose for 60s would be approximately 6×10^18^ cm^-2^ in a 6 ml-volume sample, i.e., 10^18^ atoms/ml.

### 2. Free radicals generated using CAP in aqueous solutions

**Fig 3:**
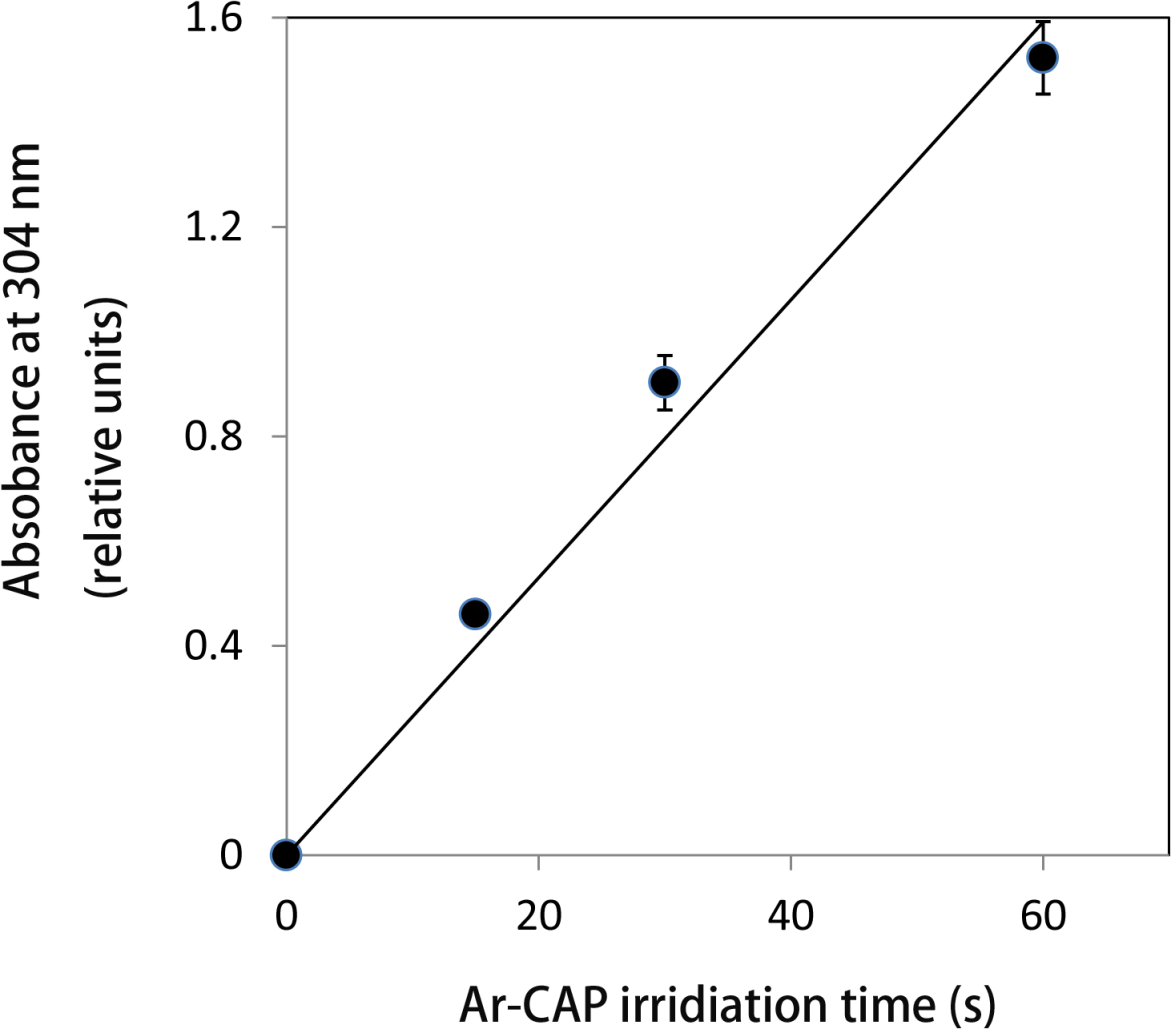
Fricke dosimetery. The effect of different Ar-CAP exposure time on the oxidation of ferrous ions to ferric ions. The oxidation increased linearly with increasing plasma irradiation time.

**Fig 4:**
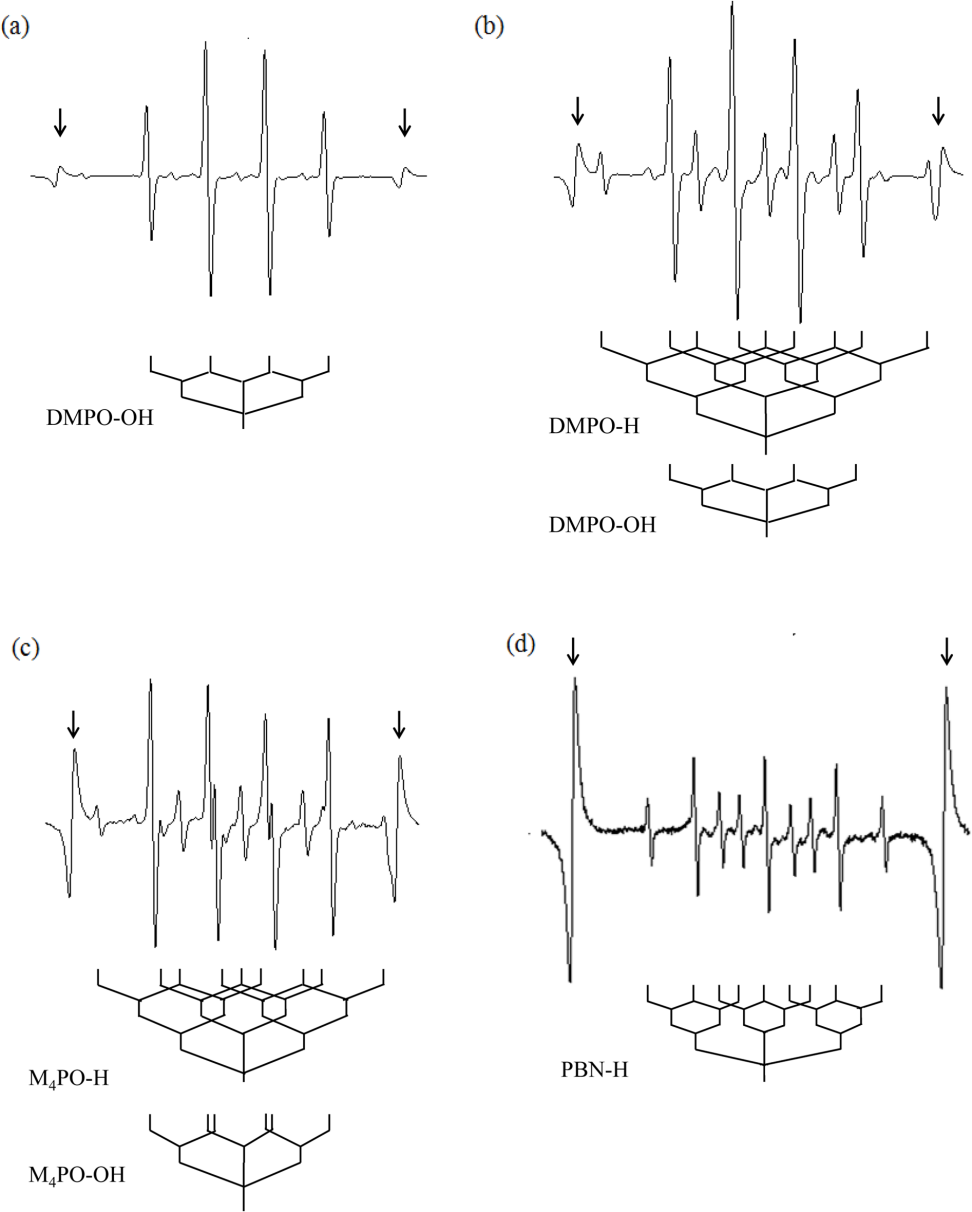
EPR spectra of spin adducts in aqueous solutions irradiated with Ar-CAP for 2 min at 8 mm distance. (a) EPR spectrum of DMPO showing a 1:2:2:1 quartet of DMPO-OH and several other small peaks due to DMPO-H. (b) DMPO aqueous solution irradiated with 5 Gy X-rays showing typical DMPO-OH and DMPO-H peaks. (c) EPR spectrum of M_4_PO-OH and M_4_PO-H adducts. (d) EPR spectrum of PBN-H adducts. Arrows indicate internal standard lines.

Plasma irradiation produces immense quantities of free radicals. The chemical activity of Ar-CAP was confirmed by Fricke dosimeter where the oxidation of ferrous ions to ferric ions increased linearly with increasing exposure time (Fig 3). However, the exact species produced by Ar-CAP are still unidentified. EPR spin trapping was employed for the detection of free radicals generated by Ar-CAP irradiation for 2 min in aqueous solutions. Ar-CAP was set at 8 mm distance between the glass tube nozzle and the surface of the sample solution. A number of spin traps with showing different characteristic spectra were used namely DMPO, M_4_PO, and PBN. To measure temperature rise, Ar-CAP irradiation in aqueous solution was performed under continuous real time temperature data recording. The temperature rise after 2 and 5 min exposure was 1.7°C ± 0.2°C (n = 4) and 2.3 °C ± 0.2°C (n = 4), respectively. When 1 ml of 10 mM DMPO aqueous solution was exposed to Ar-CAP for 2 min, an EPR spectrum consisting of a 1:2:2:1 quartet as well as other small peaks was observed. The equal nitrogen and hydrogen hyperfine coupling constants (hfcc; a_N_ = a^β^_H_ = 1.49 mT) are characteristic of the hydroxyl spin adduct of DMPO (DMPO-OH) as shown in Fig 4A. The small peaks corresponded to DMPO-H adducts showing a primary triplet (a_N_ = 1.65 mT) that is further split by two secondary hydrogens (a^β^_H_ = 2.25 mT). An EPR spectrum of DMPO-OH and DMPO-H generated by X-irradiation is shown in Fig 4B.

The formation of ·OH radicals and H atoms by Ar-CAP was further confirmed using M_4_PO and PBN. For M_4_PO, the resulting EPR spectra consisted of a primary triplet due to nitrogen (a_N_ = 1.53 mT), with each line split into a doublet by a secondary proton (a_H_ = 1.69 mT). These hyperfine coupling constants are in agreement with those reported previously for M_4_PO-OH [32,33] (Fig 4C). Other lines were analyzed as primary nitrogen triplets (a_N_ = 1.65 mT) further split by two secondary protons (a^β^_H_ = 2.17 mT), consistent with the M_4_PO spin adduct of H atoms. The EPR spectrum obtained from PBN solution (10mM) showed a primary nitrogen triplet (a_N_ = 1.67 mT) further split by two secondary protons (a^β^_H_ = 1.06 mT), consistent with the PBN spin adduct of H atoms [34] (Fig 4D). All the hyperfine coupling constants of Ar-CAP- generated radicals detected by nitrone spin trapping are shown in Table 1.

**Table 1:** Hyperfine coupling constants of Ar-CAP-induced radicals detected by nitrone spin traps.

Spin Trap	Radical	a_N_ (mT)	a^β^_H_ (mT)
**DMPO**	·OH	1.49	1.49
	H	1.66	2.25 (2H)
	C_2_H_5_O·	1.58	22.80
**M**_**4**_ **PO**	·OH	1.53	1.69
	H	1.65	2.17 (2H)
**PBN**	H	1.67	1.06

**Fig 5:**
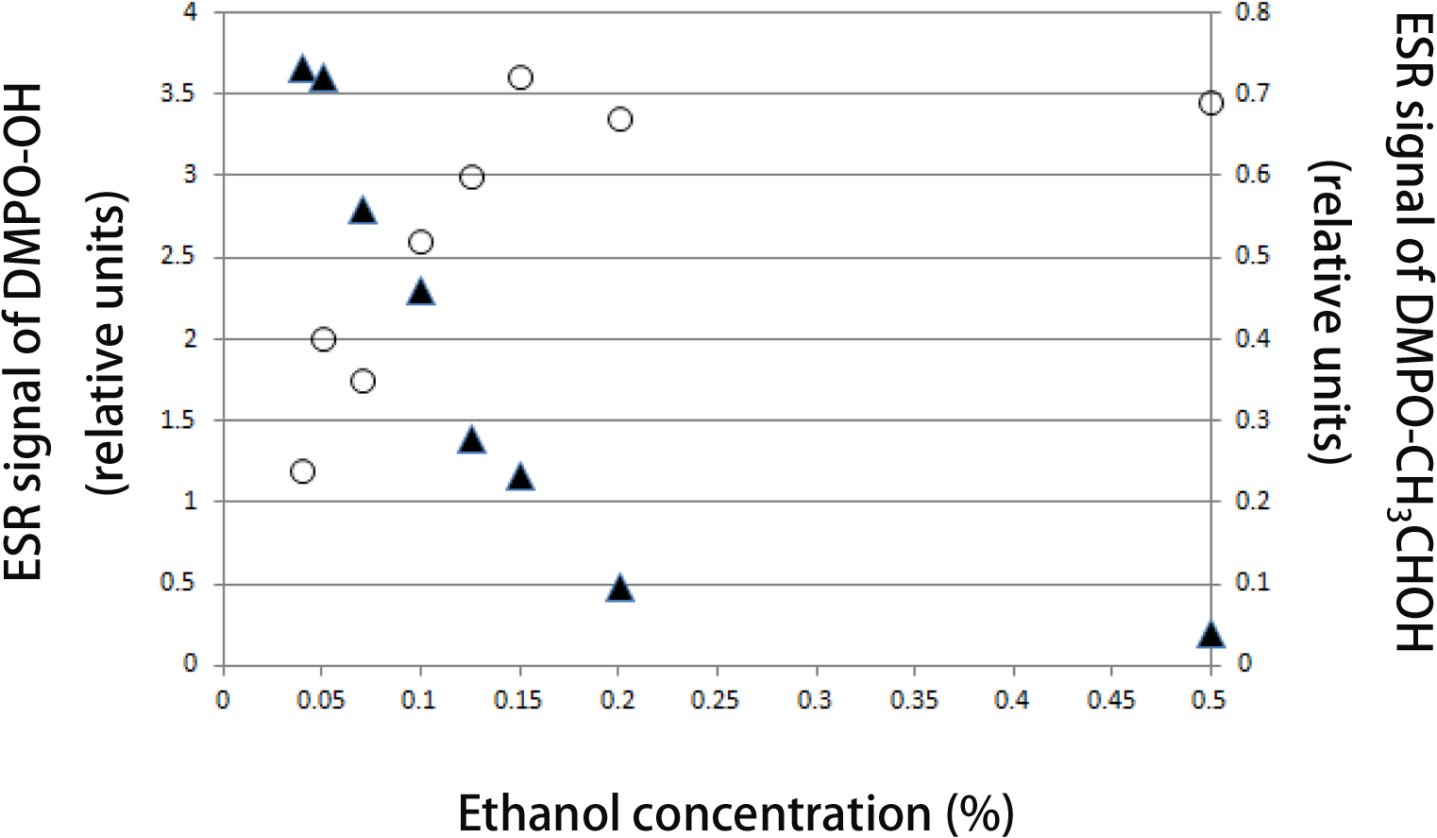
Effect of ethanol on ·OH formation. DMPO-OH adduct signal intensity decreasing with ethanol concentration with simultaneous increase in DMPO-CH_3_CHOH adduct signal intensity.

Moreover, the use of ethanol as ·OH radical scavenger further confirmed the formation of ·OH by Ar-CAP irradiation. As shown in Fig 5, the signal of DMPO-OH decreased with increasing the ethanol concentration and the signal nearly disappeared at a concentration of 0.5%. Simultaneously, the EPR signal of the DMPO-CH_3_CHOH adduct increased with ethanol concentration.

Compared with 5 Gy X-ray irradiation, Ar-CAP irradiation yielded huge amounts of ·OH radicals but smaller amounts of H atoms (Fig 4A and 4B). The amount of DMPO-OH adducts generated by Ar-CAP irradiation was also compared with that generated by UV photolysis (λ = 254 nm; at 0.45 mW/cm^2^) of hydrogen peroxide (H_2_O_2_) solution (40 mM), and the decay in the EPR spectra of DMPO-OH adducts was investigated. The half-lives of the first-order decay of DMPO-OH adducts induced by Ar-CAP irradiation and UV-photolysis of H_2_O_2_ were calculated as 6.1 ± 0.7 min and 19.2 ± 2.6 min, respectively. The rapid decay of the DMPO-OH adduct formed after Ar-CAP irradiation indicates a change in pH and/or the generation of nitric acid in the plasma-irradiated solution [35–37].

**Fig 6:**
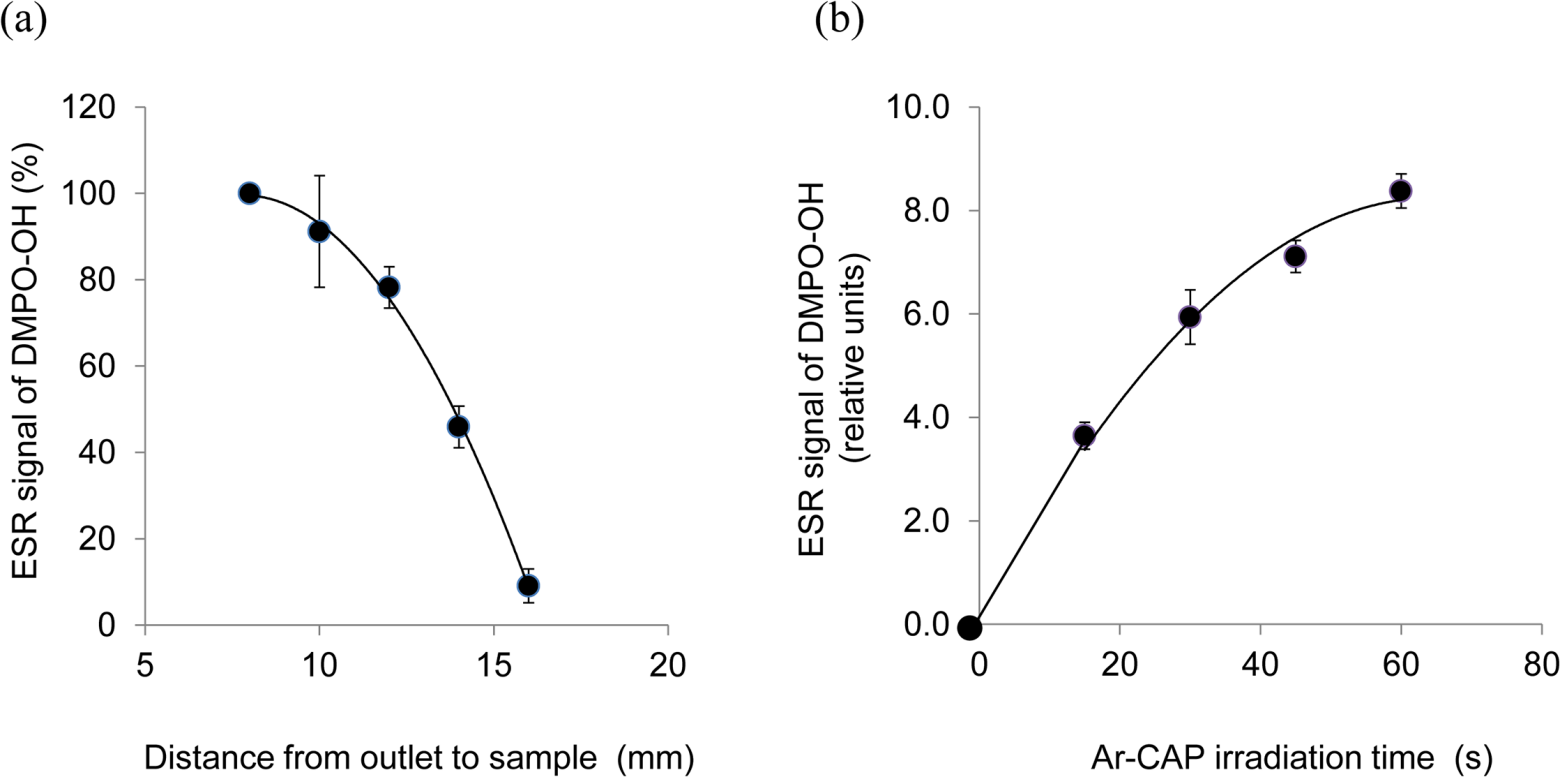
Effect of distance and exposure time on ·OH formation. (a) DMPO-OH adducts signal intensity decreases with increasing the distance from the plasma tube nozzle to sample. The yields at different distances are given as percentage from that at 8 mm considered as 100% (b) DMPO-OH adducts signal intensity increases with increasing Ar-CAP irradiation time.

When the extent of ·OH radical production was assessed at graded distances from the plasma tube nozzle, the amount of DMPO-OH adducts was found to decrease with increasing distance (Fig 6A). With the yield at 8 mm taken as 100%, 91.2 ± 12.9% yield was obtained at 10 mm, 78.2 ± 4.8% at 12 mm, 45.9 ± 4.8% at 14 mm, and 9.1 ± 3.9% at 16 mm. With respect to Ar-CAP irradiation time, Fig 6B shows that the amount of DMPO-OH adducts increased with increasing irradiation time. The yield at 30s was about 1.6 times that at 15s, and the yield at 60s was about 2.3 times that at 15s.

**Fig 7:**
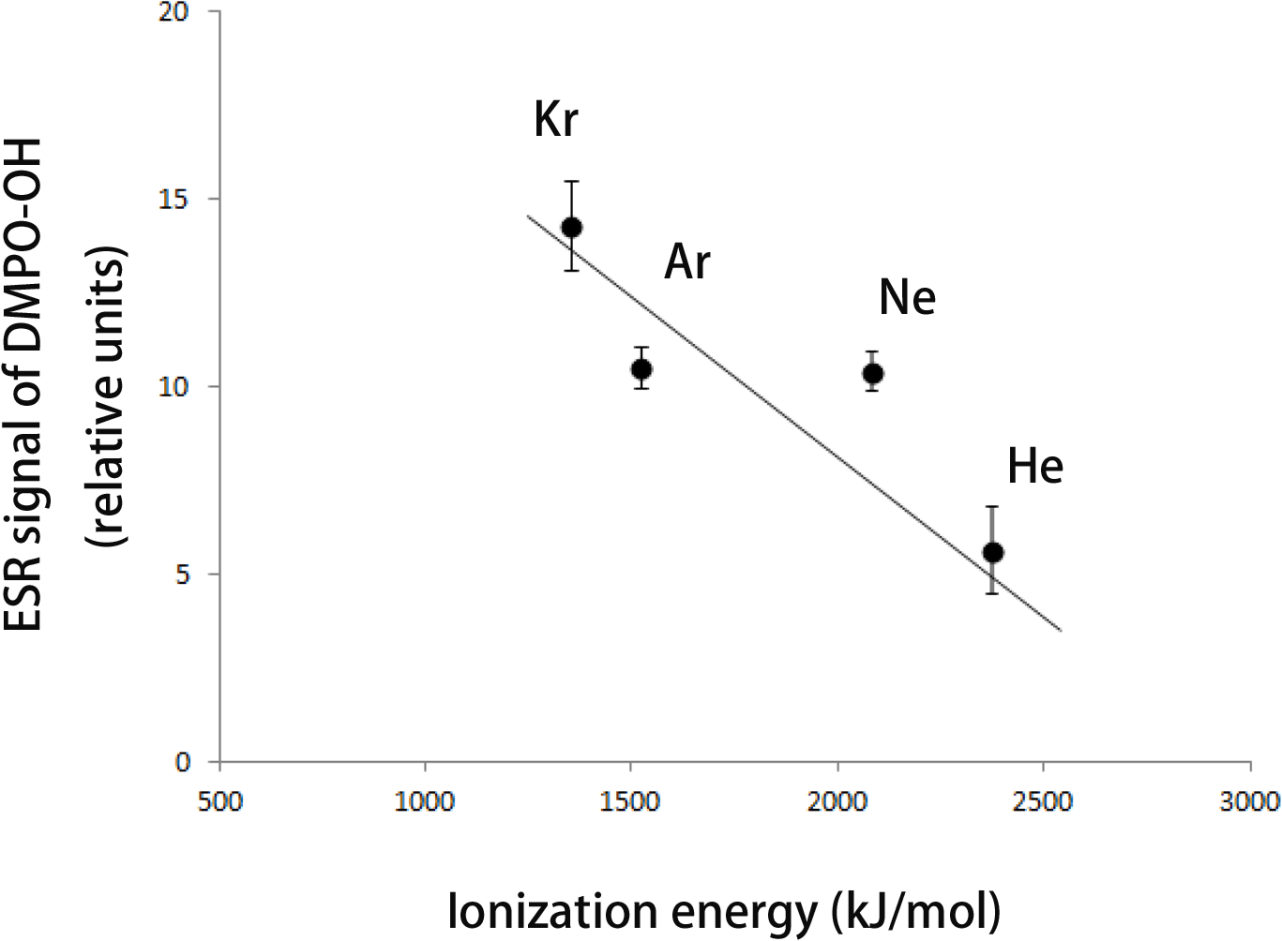
Amount of ·OH radicals produced by different rare gas—plasmas. The four rare gases krypton (Kr), argon (Ar), neon (Ne), and helium (He) were used to generate cold atmospheric plasma. The amount of^**.**^ **OH** radicals formed (indicated by DMPO-OH adduct EPR signal) correlated inversely with the ionization energy of the gas. Represented data are the mean ± SD of three independent replicates.

The effect of different rare gases on CAP-induced ·OH radical production was investigated. The yield of DMPO-OH adducts was in the order Kr > Ar = Ne > He. No plasma discharge was observed when Xe was used. When the yield of DMPO-OH adducts from different rare gas plasmas under similar conditions was plotted against the ionization energies of the rare gases used, an inverse correlation was observed (Fig 7). The NO probe cPTIO at a concentration of 200 μM was used to examine NO generated by Ar-CAP irradiation. No specific signal of c-PTIO-NO was observed in the generated EPR spectrum after a 2 min exposure. A reaction-diffusion simulation study by Ikuse et al. showed that NO generation occurs at the interface between the irradiated solution and plasma jet where it rapidly reacts with other moieties producing a variety of species [38]. Therefore, NO generation might be below the detection limit in our setup. When the nitrate/nitrite generation due to Ar-CAP irradiation was examined, the concentration in aqueous solutions was 77.3 ± 2.0 μM. It is thus concluded that Ar-CAP irradiation in water produced mainly large amounts of ·OH radicals and subsequently H_2_O_2_, owing to the rapid recombination of ·OH radicals, small amounts of H atoms, and nitrate/nitrite were also detected.

### 3. Effects of CAP on pyrimidine derivatives, L-alanine, and sodium acetate aqueous solutions

**Fig 8:**
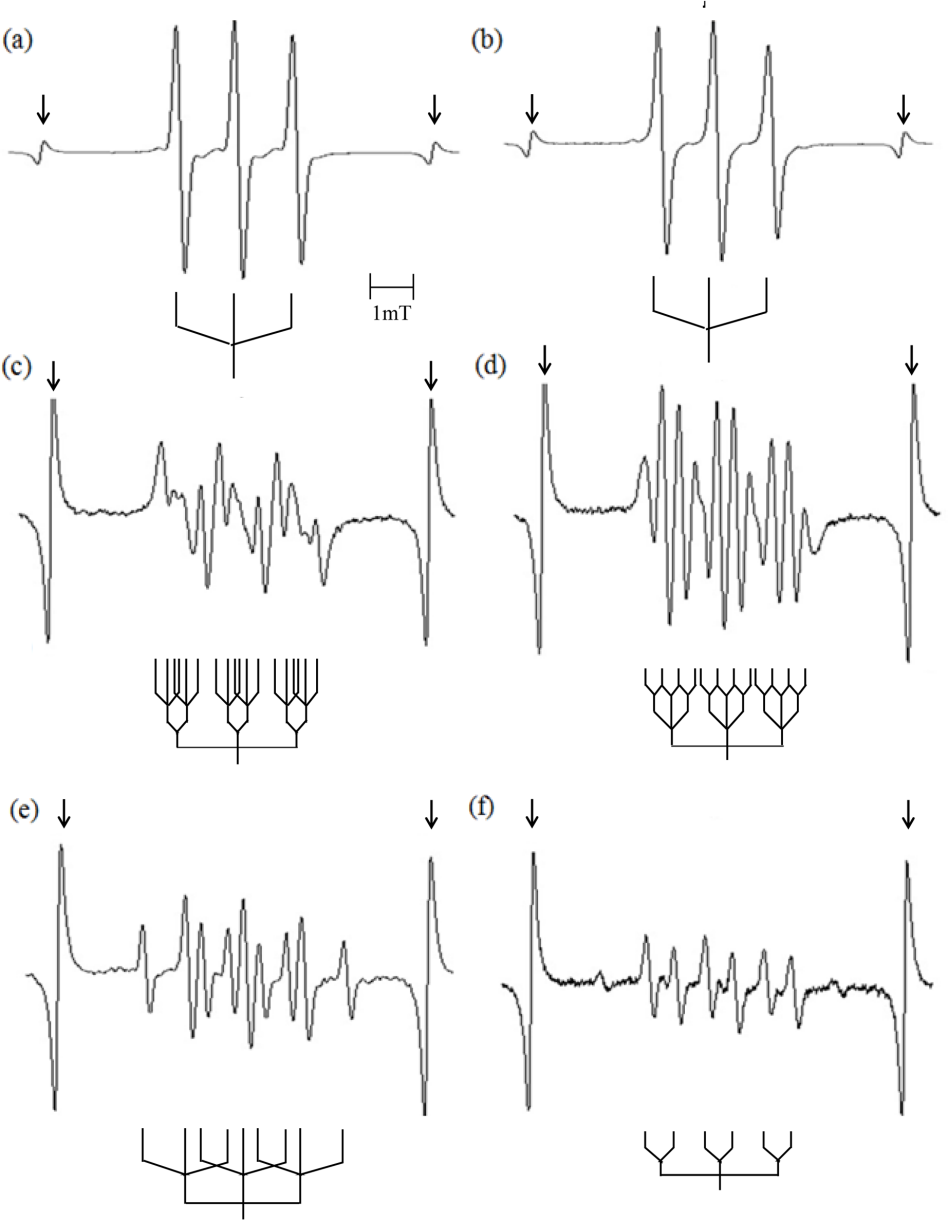
EPR spectra of DBNBS-spin adducts. (a) thymine, (b) thymidine, (c) uracil, (d) uridine, (e) sodium acetate, and (f) L-alanine. Arrows indicate internal standard lines.

The water-soluble, non-volatile, aromatic nitroso spin trap DBNBS was used for the detection of intermediate radicals formed from DNA constituents, sodium acetate, or L-alanine at high concentrations to detect specific radicals following Ar-CAP irradiation. Solutions containing thymine (20 mM) and DBNBS (5 mM) irradiated with Ar-CAP for 2 min showed an EPR spectrum of a triplet (a_N_ = 1.30 mT) which can be attributed to the spin-trapped radicals at the C-5 position most likely formed by ·OH addition (and H addition) to the C-6 position of the thymine ring (Fig 8A). The same triplet (a_N_ = 1.30 mT) was also observed for the similarly irradiated thymidine aqueous solution (100 mM) (Fig 8B). Analysis of the EPR spectra of the spin adducts indicated that there were no magnetic nuclei at the β-position of the spin adducts. In the case of uracil (20 mM) and uridine (20 mM) aqueous solutions, the EPR spectra showed secondary doublets due to a β -proton which exhibited a different hyperfine splitting, and secondary triplets due to a β-nitrogen (Fig 8C and 8D). It is speculated that the major spin adducts originated from the 6-yl radical formed by ·OH addition (and H addition) to the C-5 position of the base moiety [29,39].

Sodium acetate and L-alanine of high concentrations were irradiated with Ar-CAP to detect whether methyl radicals are formed due to pyrolysis or direct degradation, namely dissociation of C-O or C-C bond. The EPR spectrum for the sodium acetate aqueous solution at concentrations of 3 M and 5 mM DBNBS showed a primary nitrogen triplet (a_N_ = 1.37 mT) further split by two secondary protons (a^β^_H_ = 1.0 mT) (Fig 8E). The spectrum is consistent with that of the spin adduct of the acetate (·CH_2_COO^-^) radical. Unlike the case of sonication of 3.3 M sodium acetate solution for 10 min in the presence of Ar, there was no evidence of methyl (·CH_3_) radicals in Ar-CAP [29]. L-alanine aqueous solution (1 M) irradiated with Ar-CAP showed an EPR spectrum with a primary nitrogen triplet (a_N_ = 1.36 mT) further split by two secondary protons (a^β^_H_ = 1.36 mT) which indicates ·CH_2_-CH(NH_3_^+^)-COO^-^ radical formation as a result of H abstraction from the methyl group of alanine. A primary nitrogen triplet (a_N_ = 1.37 mT) further split by a secondary proton (a^β^_H_ = 0.65 mT) was also observed (Fig 8F). This is consistent with CH_3_-·CH-COO^-^ radical formation through the deamination of the alanine molecule [29]. No evidence of ·CH_3_ radicals was observed. All the hyperfine coupling constants of the Ar-CAP-generated radicals detected by a nitroso spin trap are shown in Table 2.

**Table 2:** Hyperfine coupling constants of Ar-CAP-induced radicals detected by the nitroso spin trap DBNBS

Radical	a_N_ (mT)	a^β^_N_ (mT)	a^β^_H_ (mT)
**Thymine**	1.30		
**Thymidine**	1.30		
**Uracil**	1.35	0.28	0.44
**Uridine**	1.30	0.36	0.36
**Sodium acetate**			
** CH**_**2**_ **COO** ^**-**^	1.37		1.00 (2H)
**L-Alanine**			
** CH**_**2**_ **CH(NH**_**3**_^**+**^ **)-COO** ^**-**^	1.36		1.36 (2H)
** CH**_**3**_ **-** ^**.**^ **CHCOO** ^**-**^	1.37		0.65 (H)

### 4. Intracellular ROS formation induced by Ar-CAP irradiation

**Fig 9:**
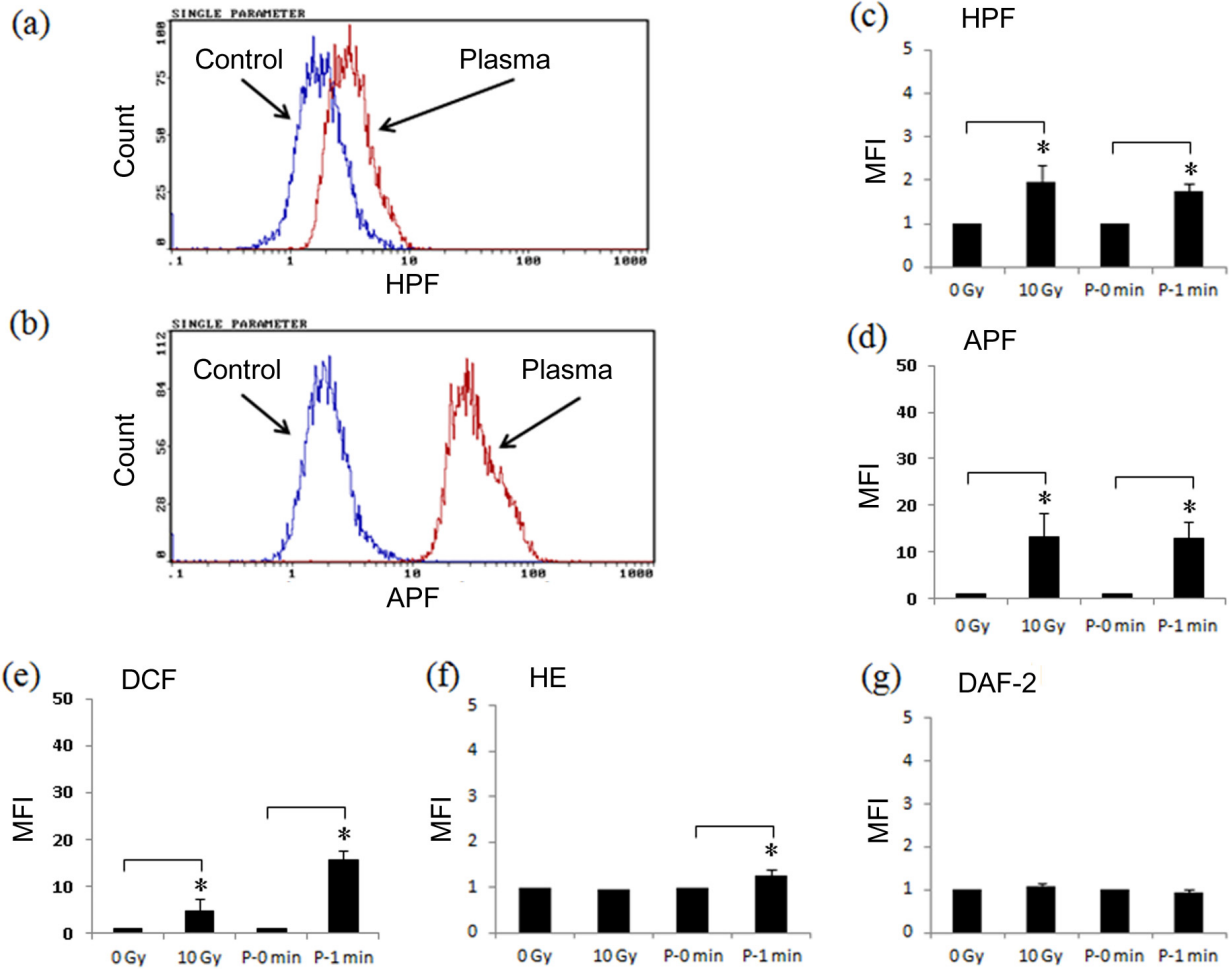
Intracellular ROS formation induced by Ar-CAP irradiation. Intracellular oxidative stress was investigated flow cytometrically using five different fluorescent probes of different affinities to a number of free radicals. U937 cells were exposed to Ar-CAP for 1 min or X-rays (10 Gy). Control samples were sham-exposed and handled exactly as irradiated cells were. The typical histograms showing the increase in fluorescence intensity following Ar-CAP (2 min) in presence of (a) HPF and (b) APF. All percentages acquired from three independent replicates are plotted in bar diagrams as mean ± SD for (c) HPF, (d) APF, (e) DCF(H), (f) HE, and (g) DAF-2. RFI, Relative Fluorescence Intensity

Ar-CAP irradiation has been shown to produce immense amounts of free radicals in aqueous solutions. In this part, the formation of free radicals within cells was investigated using five fluorescent probes, namely, HPF for the detection of ·OH and ONOO^-^; APF for ·OH, ONOO^-^, and OCl^-^; HE for O_2_·^-^; and DAF-2 for NO·. When HPF and APF were utilized to measure the intracellular oxidative stress due to Ar-CAP irradiation, the shift of distribution of fluorescence intensity was observed (Fig 9A and 9B). The quantitative data are shown in Fig 9C and 9D. A non specific fluorescent probe DCFH-DA was also used for confirming the total intracellular oxidative stress [40]. Compared with X-ray irradiation at 10 Gy, Ar-CAP irradiation for 1 min induced greater intracellular oxidative stress (Fig 9E). The increase in DCF(H) fluorescence intensity after Ar-CAP and X-ray irradiation could be explained in terms of the enhanced formation of ·OH radical and OCl^-^ as well as H_2_O_2_ resulting from the combination of two ·OH radicals. This result is in part consistent with the superiority of Ar-CAP in the formation of ·OH radicals shown by EPR spectrometry as discussed earlier. Since the increase in HPF fluorescence intensity could be mainly attributed to intracellular ·OH radicals while APF fluorescence could be attributed to both ·OH radicals and OCl^-^, then X-rays seem to generate more OCl^-^ (Fig 9C and 9D). The exact mechanism by which ·OH radicals are formed inside cells is still unclarified; however, there are three possibilities: 1) ·OH radicals may form directly inside the cells, 2) a certain potion of the enormous amounts of extracellular ·OH radicals can traverse the cell membrane, or 3) ·OH radicals may form via the Fenton reaction of H_2_O_2_.

No increase in HE fluorescence intensity was observed in the X-irradiated cells whereas very little increase was observed in cells treated with Ar-CAP for 1 min indicating the formation of small amounts of O_2_·^-^ by Ar-CAP (Fig 9F). The intracellular formation of ONOO^-^ following either treatment was unlikely because the formation of NO was excluded based on DAF-2 data (Fig 9G).

### 5. Intracellular ROS formation at isoapoptotic doses of Ar-CAP and X-ray irradiation

**Fig 10:**
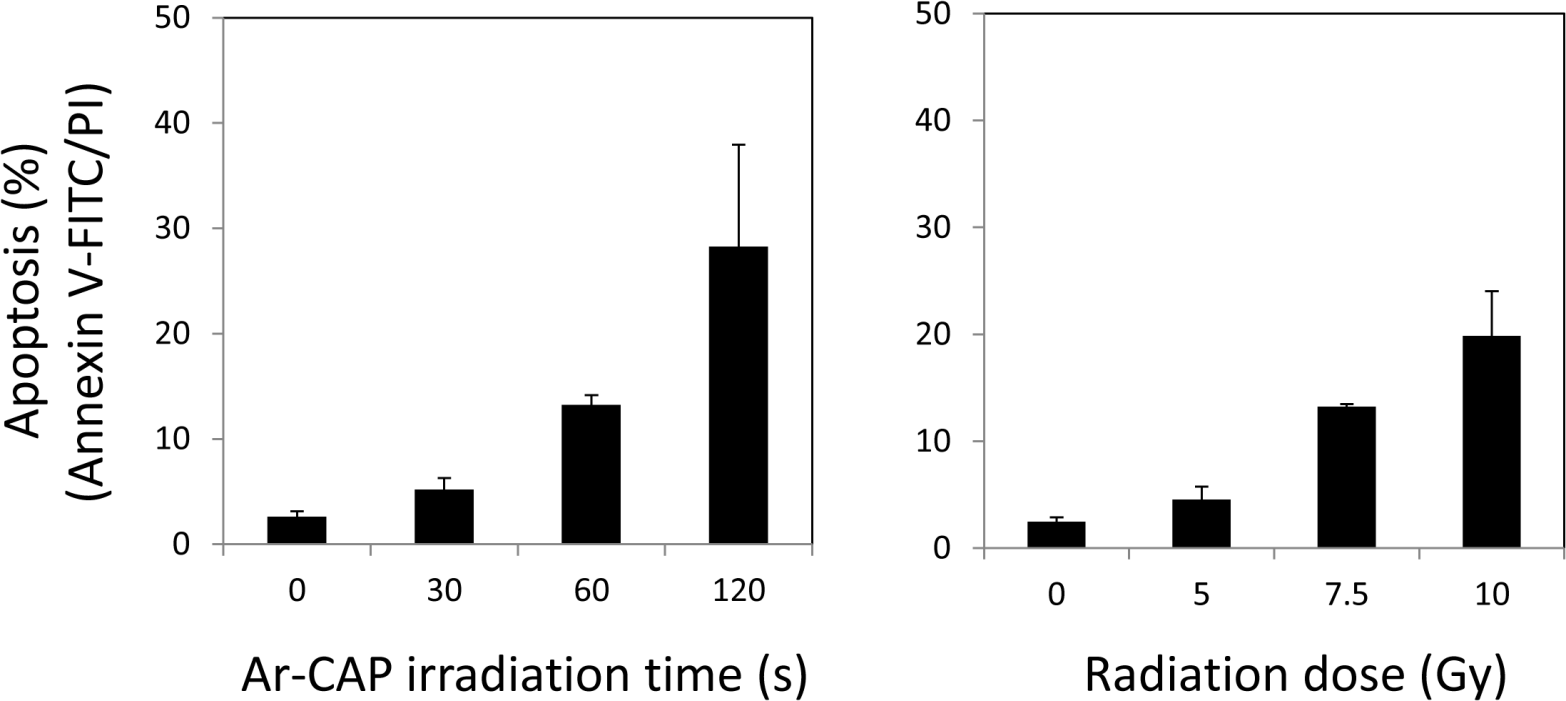
Comparison of Ar-CAP and X-irradiation with respect to apoptosis induction. The total apoptotic cell fractions in U937 cells exposed to Ar-CAP and X-irradiation investigated at 6 h post treatment by flow cytometry using annexin V-FITC and PI double staining.

Apoptosis has been associated with enhanced intracellular oxidative stress under many conditions [41,42]. Here, the total apoptosis fraction in U937 cells exposed to Ar-CAP was investigated at 6 h post treatment by annexin V-FITC and PI double staining with flow cytometry. The fractions were 5.2 ± 1.1% after 30 s irradiation, 13.3 ± 0.9% after 60 s irradiation, and 28.3 ± 9.7% after 120 s irradiation compared with 2.6 ± 0.5% in the control. In the case of X-ray irradiation, the percentages of the total apoptosis were 4.6 ± 1.2% at 5 Gy, 13.3 ± 0.2% at 7.5 Gy, and 19.9 ± 4.2% at 10 Gy compared with 2.5 ± 0.4% in the control (Fig 10). Thus, the total apoptosis fraction induced by 1 min-Ar-CAP was almost similar to that induced by 7.5 Gy X-irradiation. When the amount of DMPO-OH adducts generated by X-irradiation was measured at increasing radiation doses, the slope value was 0.03 relative unit /Gy. Consequently, if Ar-CAP treatment for 1 min produced about 6.75 relative value of DMPO-OH adducts, this would correspond to that obtained at 225 Gy of X-irradiation, i.e., Ar-CAP would generate approximately 30 times the amount of ·OH radicals produced by X-ray irradiation. Despite this striking comparison, X-ray irradiation remains more lethal than Ar-CAP irradiation and the intriguing question about ROS and their lethality still awaits answers.

## Conclusions

In this study, we have shown that Ar-CAP irradiation can generate enormous amounts of ·OH radicals and H_2_O_2_—the combination product of ·OH radicals in the aqueous phase. A theoretical calculation reveals that Ar-CAP irradiation for 1 min would be sufficient for producing ·OH radicals in aqueous solutions located at 8 mm away from the plasma tube nozzle equivalent to X-ray exposure at a dose of approximately 225 Gy. However, apoptosis level induced by Ar-CAP (1min) was almost similar to that of X-irradiation at a dose of 7.5 Gy. Thus, although Ar-CAP (1min) would create about 30 times more ·OH radicals than that formed by X-irradiation (7.5 Gy), the apoptosis-inducing efficacy of both treatments was similar reflecting the superiority of X-rays in inducing cell killing.

Our EPR-spin trapping study revealed that no pyrolysis or direct degradation radicals were formed in aqueous solutions containing non volatile solutes. Intracellularly, ·OH radicals and OCl^-^ were detected in large amounts. The latter may have resulted from the reaction between ·OH radicals and chloride anions (Cl^-^) that are abundant in cells. Unlike OCl^-^, it is not clear whether ·OH radicals were formed intracellularly or traversed from the extracellular milieu through the cell membrane. The elucidation of the mechanism by which reactive oxygen and nitrogen species are formed, and the consequent effects on cellular responses remain for future plasma research to unveil. All data are available in S1 File.

## Supporting Information

S1 File The biological effects and formation of free radicals by Ar-CAP compared to X-irradiation. Biological effects were determined based on apoptosis induction in U937 cells assessed flow cytometrically. Formation of free radicals was traced by different methods. In extracellular milieu, different spin adducts were detected by EPR spin trapping method. In presence of cells, various intracellular fluorescent probes were used with flow cytometry. Moreover, the overall chemical activity of Ar-CAP was detected with Fricke dosimeter. Data are given as mean ± SD.
(XLSX)
